# How Phenolic Compounds Profile and Antioxidant Activity Depend on Botanical Origin of Honey—A Case of Polish Varietal Honeys

**DOI:** 10.3390/molecules30020360

**Published:** 2025-01-17

**Authors:** Katarzyna Jaśkiewicz, Teresa Szczęsna, Jacek Jachuła

**Affiliations:** Apiculture Division, The National Institute of Horticultural Research, Konstytucji 3 Maja 1/3, 96-100 Skierniewice, Poland; teresa.szczesna@inhort.pl (T.S.); jacek.jachula@inhort.pl (J.J.)

**Keywords:** honey, phenolic compounds, phenolic acids, flavonoids, free radicals, total phenolic compounds, DPPH, TPC, HPLC-DAD

## Abstract

Honey contains natural biologically active compounds, and its preventive and healing properties are primarily linked to its antioxidant activity. The antioxidant properties of honey can be related to the botanical origin and content of phenolic compounds. We tested 84 honey samples from Poland, representing eight honey varieties: acacia, phacelia, buckwheat, linden, rapeseed, heather, goldenrod, and honeydew. High-performance liquid chromatography with photodiode-array detection (HPLC-DAD) was used to determine the phenolic compound composition of honey extracts. Total phenolic compounds (TPC) and DPPH radical-scavenging activity were also evaluated. We detected vanillin aldehyde, vanillic acid, caffeic acid, *p-*coumaric acid, and *trans-*ferulic acid, as well as flavonoid pinocembrin, in all honey varieties. The results of our study showed that honeys with high antioxidant activity were characterized by significantly higher total phenolic compounds content. Neither clustering method nor principal component analysis (PCA) showed clear separation of each honey variety, possibly due to high intra-variety diversities. We suppose that the variability of qualitative and quantitative phenolic compound composition within honey varieties may result from the region of origin, secondary nectar sources, and the time of harvest.

## 1. Introduction

In recent years, products with health-promoting properties have become increasingly important. Many of these products owe their benefits to compounds with antioxidant activity, particularly phenolic compounds. These compounds represent a large group of secondary metabolites synthesized in plant cells as a part of their normal growth processes or in response to various stimuli, such as damage, stress, fungal infections, or exposure to UV radiation [1].

Phenolic compounds play a crucial role in human health and are key components in dietetic and medical applications. They offer a wide range of health benefits, including the prevention of oxidative stress by scavenging free radicals and regulating enzymes that maintain oxidoreductive balance. Their effects include antioxidant, anti-inflammatory, anti-cancer, anti-atherosclerotic, spasmolytic, diuretic, detoxifying, antiarrhythmic, and hypotensive properties, as well as the ability to seal capillary vessels [2,3,4,5,6].

Phenolic compounds share a fundamental chemical structure characterized by an aromatic ring bearing one or more hydroxyl groups. These compounds can be categorized into various classes, with the main groups including flavonoids, phenolic acids, tannins, stilbenes, and lignans [7]. In honey, the most commonly found phenolic compounds include flavonoids, phenolic acids, and derivatives of phenolic acids [7,8,9,10,11]. The total phenolic content in honey varies significantly depending on its botanical origin [12,13,14]. Numerous studies have confirmed variations in the phenolic compound content among different honey varieties. Lighter honeys, such as those derived from acacia, willow, ribwort, goldenrod, clover, rapeseed, sunflower, lime, thyme, and citrus, generally have a significantly lower total phenolic content compared to darker honeys (e.g., chestnut, heather, deciduous and coniferous honeydew, and buckwheat varieties) [15,16,17,18,19,20,21].

The most common phenolic acids include gallic, caffeic, ferulic, *p-*coumaric, *p-*hydroxybenzoic, syringic, chlorogenic, ellagic, and vanillic acids [22,23,24,25,26,27].

The most abundant groups of flavonoids in honey are flavones, flavanols, and flavonols [7,28,29]. The most common flavonoids found in varietal honeys are myricetin, luteolin, quercetin, kaempferol, catechin, epicatechin, hesperetin, chrysin, pinocembrin, galangin, apigenin, naringenin, and rutin [8,24,25,27,30,31,32,33,34]. The composition and antioxidant properties of honey are primarily influenced by its botanical and geographical origin, as well as environmental factors, such as temperature and humidity, during harvest [35]. Processing and storage practices also play a smaller but notable role in affecting honey’s properties [7,9,10,11,36].

The identification of certain individual compounds or groups of phenolic compounds has been proposed as a tool for identifying the botanical and geographical origin of honey. Some phenolic compounds have been described as chemical biomarkers, including the following: quercetin for sunflower honeys; kaempferol for rosemary honeys; hesperetin for citrus honeys; naringenin, luteolin, and gallic acid for lavender honeys; myricetin, tricetin, luteolin, and kaempferol in glycoside form for acacia honeys; syringic acid, *p-*coumaric acid, ellagic acid, and *p-*hydroxybenzoic acid for heather honey; caffeic, *p-*coumaric, and ferulic acids for chestnut honey; phenylpropanoic acid for rapeseed honey; 4-hydroxybenzoic and phenylacetic acids for buckwheat honey; and rosmarinic acid for thyme honey. Quercetin, naringenin, apigenin, genistein, and protocatechuic acid can be considered potential markers for honeydew honeys [10,26,37,38,39].

However, varietal honeys may exhibit significant differences in both the qualitative and quantitative composition of their phenolic compounds, including phenolic acids and flavonoids. This is due to, i.a., geographic origin and climatic conditions [9]. As a result, it is challenging to definitively identify specific polyphenols that serve as reliable indicators for particular honey varieties [12].

This study presents a comparison of antioxidant properties and the qualitative and quantitative composition of phenolic acids and flavonoids among Polish varietal honeys. An effort was made to analyze the relationships between the botanical origin, phenolic compounds, and antioxidant properties of the honeys.

## 2. Results and Discussion

### 2.1. Botanical Origin of Honey Samples

Mellisopalynological analyses confirmed the botanical origin of the nectar honey samples and enabled their classification into seven varieties: acacia, phacelia, rapeseed, linden, heather, goldenrod, and buckwheat honey (Table 1). Among these, acacia, rapeseed, and phacelia honeys exhibited the greatest variability in specific pollen content. Honeydew honey samples (n = 17) were characterized by electrical conductivity ranging from 0.81 to 1.24 mS/cm (CV = 7.0%), with a mean value of 1.01 mS/cm.

According to melissopalynological principles, unifloral honey should contain a certain level of predominant pollen grains from a specific plant species [40]. However, requirements for classification can vary between countries. For example, buckwheat honey in some standards requires only 30% of *Fagopyrum* pollen grains, whereas in Poland, the threshold is set at 45% [41]. Such discrepancies may result in inconsistent chemical profiles in honey. Additionally, secondary nectar sources may influence the composition and variability of certain honey types [42,43].

**Table 1:** Results of melissopalynological analysis of the studied honeys.

	Specific Pollen Content				
Honey Variety	Min–Max (%)	Average (%)	Standard Deviation	Coefficient of Variation (%)	Requirements of the Minimum Percentage of Pollen According to PN-88/A-77626 “Miód pszczeli”
Acacia—Ac (n = 8)	30.5–67.0	35.6	7.4	20.8	30
Phacelia—Pha (n = 13)	47.0–91.0	61.6	14.8	24.0	45 *
Buckwheat—Bw (n = 11)	45.5–68.0	53.2	5.7	10.8	45
Linden—Li (n = 10)	25.9–45.0	37.2	4.8	12.9	20
Rapeseed—Ra (n = 10)	66.0–90.0	77.1	15.1	19.6	45
Goldenrod—Gd (n = 7)	45.0–59.2	50.0	6.1	12.2	45 **
Heather—He (n = 8)	46.7–68.0	60.1	6.3	10.5	45

* According to [44,45,46]. ** According to [47,48,49].

### 2.2. Phenolic Compounds Profile

We used the RP-HPLC technique to assess the content of vanillin aldehyde, five selected phenolic acids, and ten flavonoids in varietal honeys produced in Poland. The identified phenolic acids, vanillin aldehyde, and flavonoids in Polish varietal honeys are presented in Table 2 and Table 3.

Vanillin aldehyde, vanillic acid, caffeic acid, *p-*coumaric acid and *trans-*ferulic acid were identified and quantified in all honey varieties. It is worth noting that salicylic acid was not detected in rapeseed honey. The highest amounts of salicylic acid (mean of 394.6 μg/100 g) were identified in honeydew honey. It was suggested that salicylic acid may be a marker for honeydew honey [50,51]; however, Kuś et al. [52] did not confirm the presence of the acid in all honeydew samples from Poland. The *p-*coumaric acid content was in a range of 75.9–382.8 μg/100 g and was the highest in heather honey. Much higher but strongly variable (28–1566 μg/100 g) content of *p-*coumaric acid in heather honey from Silesia region was found by Jasicka-Misiak et al. [9]. The content of caffeic acid was in a similar range as that found by Puścion-Jakubik et al. [53] for buckwheat, heather, and honeydew honey. However, the authors calculated more than 10 times higher values for linden honey. The highest content of *trans-*ferulic acid (mean >200 μg/100 g) was detected in heather and honeydew honeys. In contrast to our study, Puścion-Jakubik et al. [53] found the highest amounts of this acid in linden honey (ca. 2000 μg/100 g on average). From all the analyzed samples, significant amounts (120.8–280.2 μg/100 g) of vanillic acid were found in linden, goldenrod, heather, and honeydew honeys. On the contrary, Farkas et al. [54] obtained nearly three times lower values for linden and goldenrod honeys from Hungary.

Schanzmann et al. [55] suggested vanillin as a marker for linden and acacia honey. We detected especially high, but also variable, amounts of vanillin in linden honey (mean 1354.1 μg/100 g, RSD = 65.2%). However, only small amounts of vanillin (mean 36.6 μg/100 g) were detected in acacia honey in our study.

Pinocembrin was identified in all honey varieties at a similar content level (mean 74.5–164.4 μg/100 g). This flavonoid is quite commonly found in many plant species, and it has been identified in different honey varieties, as well as in propolis [56]. Hesperidin is considered as a marker compound for citrus honey [57]. However, it was detected in all samples in our study, except acacia honey, and its concentration was highest in rapeseed honey. In this study, rutin was detected in all honey varieties except heather honey. Absence of rutin was also shown in other studies on Polish honeys [58] and Latvian honeys [59]. Our study revealed that quercetin, hesperetin, and luteolin were not commonly found in varietal honeys, and, when detected, their concentrations did not exceed 35 µg/100 g. The sole exception was linden honey, where a high concentration of quercetin (mean = 223.4 µg/100 g) was observed. Hesperetin is less common in nature than its glycoside form, hesperidin. The presence of hesperetin may be a result of hydrolysis of hesperidin. However, we did not study the content or activity of enzymes responsible for the conversion of these compounds. Also, it is known that extraction in an acidic environment may degrade hesperidin and possibly increase the content of hesperetin [60]. In contrast to our study, according to Socha et al. [30], the main flavonoids in Polish honeys are hesperetin, kaempferol, and quercetin. Quercetin, hesperetin, and luteolin were found in acacia honey from Croatia [61]. Moreover, quercetin was suggested as a marker compound for acacia honey in the study on Hungarian honeys [54]. The same study showed the absence of quercetin in linden honey and the presence of kaempferol in acacia honey, which is entirely contrary to our findings. Unlike in our study, Chinese [62] and Kazakhstani [63] buckwheat honey contained chrysin. Wilczyńska [12] identified kaempferol in Polish honeydew honey and both kaempferol and chrysin in buckwheat honey.

### 2.3. Total Phenolic Content (TPC) and Antioxidant Activity

The results of the total phenolic content analysis in the different honey varieties are presented in Table 4. The mean content of phenolic compounds ranged from 17.2 GAE/100 g to 123.5 mg GAE/100 g. The botanical origin of the honey influenced the total content of phenolic compounds, and dark honeys (buckwheat, heather, and honeydew honey) had significantly higher TPC than light honeys. This is consistent with results for other Polish honeys: TPC was found to be as low as 13.5 for rapeseed honey and as high as over 180 for buckwheat and multifloral dark honeys [53,64]. Kędzierska-Matysek et al. [14] found, similar to results of our study, low values of TPC in rapeseed (15.8 mg GAE/100 g) and acacia (18.7 mg GAE/100 g) honeys. Pentoś et al. [21] found that, for buckwheat honey, TPC was 334.0 mg GAE/100 g; for heather honey, 242.2 mg GAE/100 g; and for honeydew honey, 183.9 mg GAE/100 g. Surprisingly, some papers reported much lower TPC values for all honey varieties tested. According to Socha et al. [30], the total content of phenolic compounds in Polish honeys ranged from 4.5 mg for rapeseed honey to 15.0 mg GAE/100 g for buckwheat honey. Similarly, in the study of Wesołowska and Dżugan [15], low values for the TPC were obtained, which ranged from 7.6 to 10.5 mg GAE/100 g for rapeseed honey (average 9.2 mg GAE/100 g) and from 19.4 to 32.5 mg GAE/100 g for buckwheat honey (average 28.1 mg GAE/100 g). Also, in Slovenian honeys [65], TPC values were in the range from 4.5 mg GAE/100 g (acacia honey) to 24.1 mg GAE/100 g (for honeydew honey). These discrepancies suggest the effect of geographic origin (even on the regional scale) and seasonality (different weather conditions between seasons) on the TPC. It also cannot be overlooked that obtained TPC values depend on the measurement process itself, e.g., the ratio between honey sample and Folin–Ciocâlteu reagent (1:10 in [15,17,65], 1:2 in [21]; and 1:5 in [22,30], and the present paper), or even on the preparation of a calibration curve [66].

In terms of antioxidant activity, buckwheat honey and honeydew honey showed the highest values (mean = 89.4% and 91.7%, respectively), contrasting with acacia and phacelia honey (about 18%). In the case of other honeys (goldenrod, rapeseed, linden, and heather), a moderate deactivating activity against the DPPH radical was observed (25.1–49.6%). This is in agreement with other studies, which evidenced that, generally, dark honeys (e.g., buckwheat and honeydew honeys) were more effective in neutralizing the DPPH radicals than light honeys (e.g., acacia and phacelia honeys) [13,15,53,65,66,67,68,69]. However, in studies conducted by Socha et al. [30] on Polish honeys from the Małopolska (Lesser Poland) region, the antioxidant activity of buckwheat (46.4%) and nectar–honeydew honey (34.6%) was about two times lower than that obtained in the present study. Low values of antioxidant activities were also reported in a study on honeys from Southeastern Poland conducted by Wesołowska and Dżugan [15] (on average, lime honey—12.4%; rape honey—13.6%; goldenrod honey—16.9%; nectar–honeydew honey—20.6%; heather honey—28.3%; and buckwheat honey—48.6%). On the other hand, Wilczyńska [70] determined antioxidant activity to be 100% for heather and buckwheat honey collected from different regions of Poland. The abovementioned differences suggest a strong influence of the season on antioxidant activity or even the importance of location of apiary from which honey is obtained. In this study, we evaluated the antioxidant activity of honey based on only one assay, DPPH, which is based on an electron transfer. This may create some bias when trying to compare the results between honey varieties. To obtain a wider and more reliable picture of the antioxidant capacity, additionally, proton transfer-based assay is also used [71].

As shown in Table 5, in terms of correlation between particular phenolic compounds and antioxidant activity, no significant or only weak correlations (either positive or negative) were found. Some authors suggest that, while phenolic compounds may be responsible for the antioxidant activity of honey, other compounds (e.g., proteins, enzymes, ascorbic acid, and carotenoids) can also boost the activity toward free radicals [72,73]. However, the antioxidant properties were strongly positively (r = 0.89) correlated with the total content of phenolic compounds (TPC). This is in line with Kuś et al. [74] and Majewska et al. [75], who reported an even higher correlation coefficient (r = 0.94–0.98) between antioxidant activity (expressed as DPPH radical scavenging activity) and TPC. Strong positive correlations (r > 0.7) were also reported between antioxidant activity (ABTS) and TPC, as well as between reduction capacity (FRAP) and TPC by Kędzierska-Matysek et al. [14]. In our study, antioxidant activity was not correlated with specific pollen content in nectar honeys, but a moderately positive correlation with electrical conductivity in honeydew honeys was calculated. While pollen content can be correlated with TPC, it does not necessarily have to be correlated with antioxidant activity, as shown by Halagarda et al. [32] for buckwheat pollen. A strong correlation between radical-scavenging capacity and electrical conductivity was reported by Vela et al. [76].

### 2.4. Multivariate Analyses

**Figure 1:**
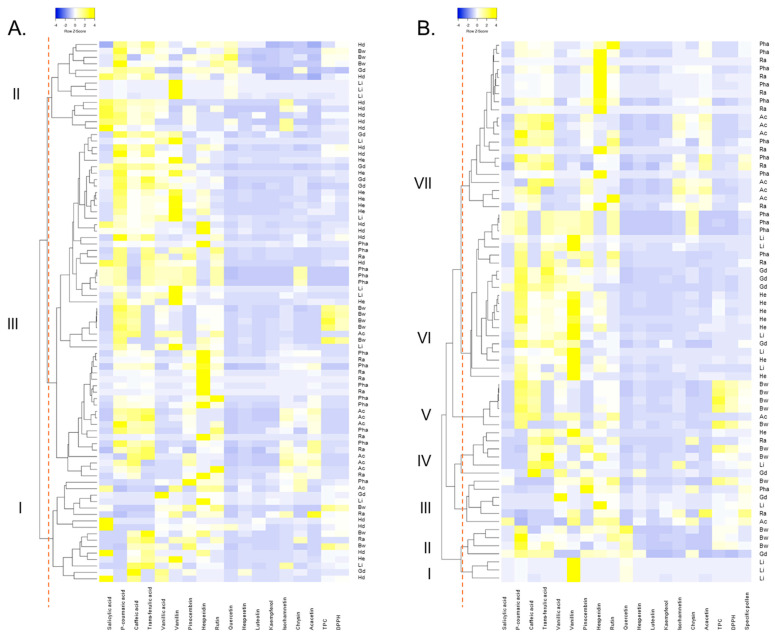
Dendrogram of hierarchical cluster analysis made for all varietal honeys (**A**) and for nectar honeys only (**B**). TPC—total phenolic content; DPPH—antioxidant activity against DPPH radical.

The results of hierarchical cluster analysis were visualized as dendrograms combined with heatmaps. As shown in Figure 1A, when all samples were analyzed together, three main clusters were formed: (I) samples with low *p-*coumaric acid content at the bottom part of the dendrogram; (II) samples with high *p-*coumaric acid quantities and moderate-to-high content of salicylic acid at the top part of the dendrogram; (III) rest of the samples, which are further divided into sub-clusters according to the content of caffeic acid, vanillic acid, and vanillin. Yet, no clear pattern of grouping according to the botanical origin was obtained. Analysis performed only for nectar honeys (Figure 1B) enabled the formation of seven clusters: (I) samples with high content of vanillin and average values for all other parameters; (II) samples with high quantities of *p-*coumaric acid and quercetin; (III) samples with low quantities of *p-*coumaric and caffeic acids; (IV) samples with moderate-to-high content of caffeic and *trans-*ferulic acids; (V) samples with moderately high *p-*coumaric and caffeic acids content, as well as TPC and antioxidant activity; (VI) samples with high quantities of *p-*coumaric and *trans*-ferulic acids and vanillin, containing most of the heather and goldenrod honey samples; and (VII) samples with moderately high content of *p-*coumaric, caffeic, and *trans*-ferulic acids cluster containing only light honeys (most of the acacia, rapeseed, and phacelia honey samples). Also in this analysis, no obvious grouping pattern of samples in terms of the botanical origin of honey was achieved.

**Figure 2:**
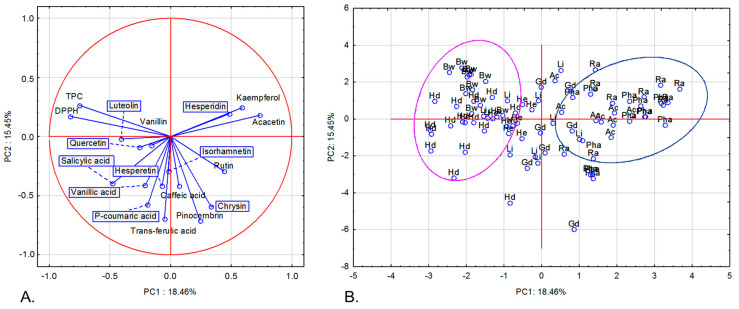
Projection of variables (**A**) and cases (**B**) in two-factor plane (PC1 × PC2). Analysis was made for nectar and honeydew honey samples. TPC—total phenolic content; DPPH—antioxidant activity against DPPH radical.

Principal component analysis (PCA) is a statistical technique commonly used to reduce the number of dimensions in large datasets. In this study, PCA was utilized to analyze 84 honey samples using 18 variables and identify those with similar characteristics. Seven principal components with eigenvalues exceeding 1 (according to the Kaiser criterion) explained 74.51% of the total variance (Supplementary Table S1). Figure 2A visualizes the projection of variables on a two-factor plane (PC1 × PC2). The first component (PC1), explaining 18.46% of variability, is correlated positively, e.g., with acacetin and negatively with phenolic acids; some flavonoids, e.g., quercetin and luteolin; TPC; and DPPH (Supplementary Table S2). The second component (PC2), explaining 15.45% of variability, is negatively correlated with most of the variables. Two groups are separated in PC1 × PC2 projection (Figure 2B). The first one, located on the left side of the graph, is composed almost exclusively of dark honeys that have negative values for PC1, implicating high TPC content and antioxidant activity and low content of acacetin. The second group, on the right side of the graph, is composed of light honey and contains most of the acacia, phacelia, and rapeseed honey samples. These samples are characterized by a high content of acacetin and low values of TPC and antioxidant activity.

**Figure 3:**
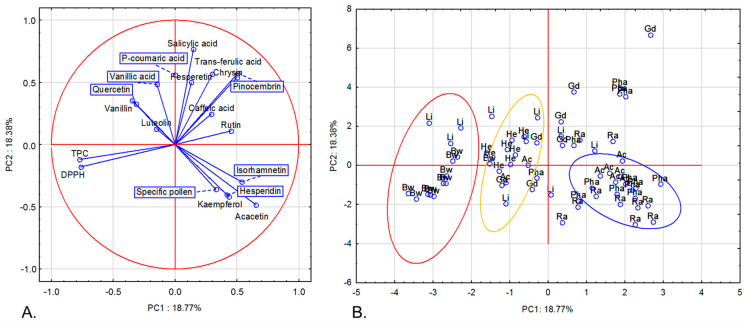
Projection of variables (**A**) and cases (**B**) in two-factor plane (PC1 × PC2). Analysis was made only for nectar honey samples. TPC—total phenolic content; DPPH—antioxidant activity against DPPH radical.

We also applied PCA on the set of nectar honey samples (detailed results: Supplementary Tables S3 and S4). Briefly, three groups were distinguished (Figure 3). The group on the left side of the plot is composed of almost all of the buckwheat honey samples and is highly negatively influenced by PC1 (Figure 3B). This can be explained by its high content of TPC and antioxidant activity. The second group, in the middle of the plot, contains mainly samples of heather honey. This assignment is possibly due to the high content of vanillin and vanillic acid, but moderate TPC and antioxidant activity. The third group contain acacia, phacelia, and rapeseed honey samples, whose placement results from low TPC and antioxidant activity, but high content of acacetin and specific pollen.

Some authors showed that, using PCA, separation of honey samples depending on their botanical origin is possible. Vazquez et al. [77] obtained partial separation by relying solely on phenolic compound content. Kędzierska-Matysek et al. [14] confirmed the distinctiveness of Polish honeys based only on their antioxidant activity and content of phenolic acids and flavonoids. Also, Ciucure and Geană [17] showed the usefulness of phenolic compounds and antioxidant activity parameters as a matrix for differentiating honey varieties in PCA. However, Oroian and Sorina [78] used both phenolic compound content and physicochemical properties in the PCA.

In this study, we attempted to define relationships between antioxidant activity, phenolic compound profiles, and the botanical origin of honey. While for some phenolic compounds, statistically significant differences were found, usually high intra-variety variabilities were also calculated. We suggest that they affected the multivariate analyses and hindered clear separation of honey varieties according to the properties studied. Variability in the composition and content of individual phenolic acids in honey is influenced by many factors. Climatic conditions, seasonality, and geographic origin can cause differences in the chemical composition even within the same honey variety [79,80,81,82]. The content of phenolic compounds depends not only on the type of nectar and honeydew forage, but also on the season and location of the apiary (local environmental factors) [82], which are probably the most important factors in our study. The palynological analysis performed in the present study made it possible to classify the honey samples as varietal honeys according to the minimal level of the content of specific pollen required. However, we do not know what the proportion of nectar from other plants or honeydew in the studied varietal honeys was. Phenolic compounds enter honey from nectar, pollen, and propolis [14]. The differences in the composition of phenolic compounds in varietal honeys may therefore depend on the variation in food sources accompanying the main forage occurring in the location of apiaries. According to some authors, in temperate regions, the flavonoids found in honey come from the resinous substances of poplar buds diffusing into honey through contact with propolis found in the hive. In the literature, it can be found that more than 90% of the flavonoids found in honey are derived from propolis (e.g., pinobanksin, pinocembrin, and chrysin) [10,41]. The content of phenolic compounds in varietal honeys may therefore be determined by differences in the phenolic compound composition of propolis, which in turn depends on its geographic origin [12,83]. It should not be ignored that a honey’s chemical profile may also change over time of storage, even independently of storage conditions. For example, some flavonoid glycosides can be sensitive to hydrogen peroxide, which is present in honey [84].

## 3. Materials and Methods

### 3.1. Reagents

DPPH (2,2-diphenyl-1-picrylhydrazyl); formic acid; vanillin; and standards of phenolic compounds, including phenolic acids such as gallic, caffeic, vanillic, salicylic, *p-*coumaric, and *trans-*ferulic acid; and flavonoids (hesperidin, quercetin, kaempferol, isorhamnetin, acacetin, hesperetin, pinocembrin, and chrysin) were purchased from Sigma-Aldrich (Poznań, Poland). Rutin trihydrate was purchased from Fluka (Poznań, Poland). Potassium hexacyanoferrate (II), K_4_Fe(CN)_6_ × 3H_2_O; and zinc acetate, Zn(CH_3_COO)_2_ × 2H_2_O, used for the preparation of Carrez reagents I and II, ethyl alcohol, Folin–Ciocâlteu reagent, and anhydrous sodium carbonate were purchased from POCH (Gliwice, Poland). J.T. Baker (Dventer, The Netherlands) supplied methanol for HPLC analyses and BakerBond C18-SPE 500 mg/6 mL solid-phase extraction columns. All reagents were of analytical-grade purity.

### 3.2. Honey Samples

Varietal honey samples were collected directly from beekeepers and from apiaries of the Apiculture Division, the National Institute of Horticultural Research. Samples were obtained from different regions of Poland (Lublin Upland, Upper Silesia, Lower Silesia, Pomerania, Mazovia, and Masuria). Samples were stored at +4 °C until they were analyzed. The botanical origin of the honey was determined on the basis of organoleptic examination, and the percentage of the main pollen content was measured via microscopic pollen analysis, following the recommendations of the International Commission for Bee Botany and the International Commission for Honey [85], in accordance with the Polish Standard for Honey [86] and the Regulation of the Minister of Agriculture and Rural Development of 14 January 2009 on methods of analysis for assessing the value of honey [87]. For this purpose, an optical microscope (OLYMPUS CX33, Olympus, Tokyo, Japan) was used, together with the OLYMPUS QuickPhotoCamera computer image analysis system (Olympus, Tokyo, Japan). The results were converted into percentages. Honeydew honeys were classified on the basis of electrical conductivity analysis using a WTW inoLAB Cond 700 conductivity meter (WTW, Weilheim, Germany), in accordance with the Regulation of the Minister of Agriculture and Rural Development of 14 January 2009 on methods of analysis for assessing the value of honey [87] and the presence of honeydew indicators (algae and Ascomycota cells) in the honey deposit visible under the microscope. Three replicates were performed for each honey sample. The result was given in mS/cm. The following honey varieties were identified: acacia honey, rapeseed honey, linden honey, heather honey, buckwheat honey, phacelia honey, goldenrod honey, and honeydew honey.

### 3.3. Isolation of Phenolic Compounds from Honey Using SPE Method

For the isolation of phenolic compounds, 5 g of honey was weighed out and dissolved in a small amount of 1% formic acid in water. The solution was then transferred quantitatively to a 50 mL volumetric flask, 0.5 mL each of Carrez reagent I (0.15 g/mL K_4_Fe(CN)_6_ × 3H_2_O) and Carrez reagent II (0.30 g/mL Zn(CH_3_COO)_2_ × 2H_2_O) was added, the substances were mixed thoroughly, and the volumetric flask was made up to the mark with 1% formic acid water. The resulting honey solution was filtered through a tissue filter for qualitative analysis (type 11A, diameter 125 mm, Bionovo, Legnica, Poland).

**Figure 4:**
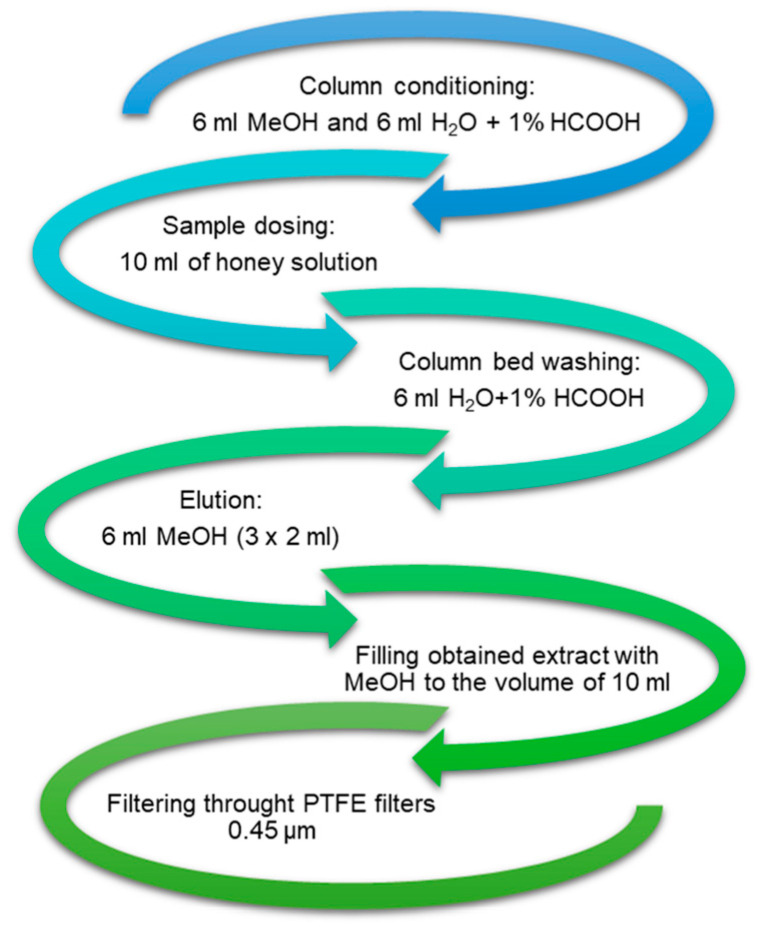
Stages in the extraction of phenolic compounds from honey using the SPE technique.

The obtained filtrate was used to isolate phenolic compounds by solid-phase extraction (SPE) using columns packed with C18-SPE octadecyl sorbent (5 mg/6 mL, Bakerbond) and a vacuum kit (J.T. Baker). Figure 4 shows the successive steps in the extraction of phenolic compounds from honey using the SPE technique.

### 3.4. HPLC-DAD Analysis of Phenolic Compounds

Analysis of phenolic acids and flavonoids was performed using an HPLC chromatograph (Shimadzu, Poland) equipped with a photodiode array detector (SPD-M20A), according to the method described by Silici et al. [88]. The separation was performed on a Synergi Fusion-RP 80A column (250 × 4.6 mm, pore size 4 μm, Phenomenex, Torrance, CA, USA) using a mobile phase of 1% formic acid (solvent A) and 100% methanol (solvent B), with the program as follows: 84% solvent A and 16% solvent B at the initial stage, 10% solvent A and 90% solvent B at 50 min, and 84% solvent A and 16% solvent B at 51–62 min. The column temperature was kept at 40 °C.

The flow rate of the mobile phase was 1 mL/min. Identification of phenolic acids and flavonoids in honey samples was performed via a comparison of chromatograms and retention times (RTs) of individual analytes with standards (Supplementary Tables S1 and S2). Quantitative analysis of flavonoids and phenolic acids was performed using the external standard method (calibration curve method). Vanillic acid, vanillin, hesperidin, and rutin were quantified against their standards at 270 nm. *p-C*oumaric acid, salicylic acid, and hesperetin were quantified at 300 nm. Caffeic acid, *trans*-ferulic acid, quercetin, kaempferol, luteolin, and acacetin were quantified at 320 nm. Each measurement was performed in three technical replicates, and the mean value was calculated.

### 3.5. Determination of the Total Phenolic Compounds Content Using Folin–Ciocâlteu Reagent (TPC)

The determination of total phenolic compounds in varietal honeys by the Folin–Ciocâlteu spectrophotometric method was carried out according to the method of Meda et al. [89], with slight modifications. In the first step, 5 g of honey was dissolved in a small amount of water, combined with 0.5 mL of each Carrez I and Carrez II reagents, and filled up to the final volume 50 mL. The solution was then placed on a mechanical shaker for 30 min. After filtering through a soft filter paper, 0.5 mL of the honey solution was mixed thoroughly with 2.5 mL of 10% Folin–Ciocâlteu reagent. After 5 min, 2 mL of 0.7 M Na_2_CO_3_ solution was added, and the mixture was incubated for 2 hours in the dark at room temperature. The absorbance of the resulting solutions was measured against a blank at 760 nm, using a spectrophotometer (SPEKORD 200 Analytik Jena, Jena, Germany). The calibration curve was determined from known concentrations of gallic acid in the range from 0 to 200 mg/L (at intervals of every 50 mg/L; calibration curve: y = 0.0113 + 0.0263; R^2^ = 0.999). The total phenolic content in honey samples was expressed as mg of gallic acid equivalents (GAE) per 100 g of honey. Three technical replicates were performed for each measurement.

### 3.6. Determination of Antioxidant Activity Against the DPPH Radical

The antioxidant activity of honey was determined spectrophotometrically, using the stable radical 2,2-diphenyl-1-picrylhydrazyl (DPPH) scavenging assay, as described in the literature [89], with slight modifications. Briefly, 2.5 g of honey was dissolved in 50 mL of methanol. From the honey solution, 0.75 mL was taken and mixed with 1.5 mL of a solution of DPPH radical in methanol at a concentration of 0.02 mg/mL. At the same time, a blank test was performed in which, instead of the honey solution, 0.75 mL of methanol was measured. The solutions were incubated for 30 minutes at room temperature, with no exposure to light. Absorbance measurements were performed in three technical replicates on a SPECORD 200 Analytic Jena spectrophotometer at λ = 517 nm against methanol as a reference sample. Antioxidant activity (%) was calculated using the following formula:

A(%) = (A_0_ − A_m_)A_0_ × 100

where A_0_—absorbance of a solution of the DPPH radical (blank test); and A_m_—mean absorbance value of the honey solution tested.

### 3.7. Statistical Analysis

If the calculated content of the determined compound was lower than the limit of detection (LOD), a value of LOD/2^0.5^ was used for calculations [90,91]. The initial number of varietal honey samples in this study was 112. The samples for which the values of the studied traits were below Q_1_ − 1.5 IQR or above Q_3_ + 1.5 IQR were discarded from each data subset (honey variety). In total, 84 samples were subjected to statistical analyses.

A comparison of the content of phenolic compounds between honey varieties was performed. Since the assumptions of normality of distribution (Shapiro–Wilk test) were not met, the non-parametric Kruskal–Wallis test was used. Spearman’s rank correlation coefficient was used to describe the correlation between phenolic compound content, specific pollen content or electrical conductivity, and antioxidant activity. Cluster analysis was applied to obtain hierarchical relationships and visualized as dendrograms combined with heatmaps. To demonstrate the variability in phenolic compound composition among honey varieties, the principal component analysis (PCA) was used. Prior to performing multivariate analyses, the data were normalized. Statistical analyses were performed using Statistica 13.1 (Statsoft, Kraków, Poland).

## 4. Conclusions

Our findings showed that the differences in particular phenolic compound content amongst the tested honey samples can be attributed to the botanical origin. The highest amounts of salicylic acid were found for honeydew, and the highest concentrations of vanillin were recorded for linden honey. Quercetin, hesperetin, and luteolin were found in several honey varieties, but only in small amounts. The TPC and antioxidant activity were dependent on honey variety, with the highest values obtained for dark honeys, such as buckwheat, heather, and honeydew, in comparison with light honeys, such as rapeseed, acacia, and phacelia. However, using multivariate analysis, no clear pattern in the phenolic profile was obtained in this study. Dark and light honeys were separated using PCA. When taking into account only nectar honeys, additionally, buckwheat and heather honey were positioned in separate groups. We assume that, in the rest of honey samples, too-high variability in the phenolic compound profile, TPC, and antioxidant activity hindered clear separation according to honey variety. It can probably be explained by different weather conditions between seasons in which honey was collected and regional-scale differences in environmental conditions. We also emphasized the possible impact of secondary nectar sources in shaping phenolic compound profile and antioxidant activity.

## Figures and Tables

**Table 2:** Content of phenolic acids and vanillin aldehyde in Polish varietal honeys.

	Honey Variety	Salicylic Acid	*p-*Coumaric Acid	Caffeic Acid	*trans-*Ferulic Acid	Vanillic Acid	Vanillin
Min–max (µg/100 g)	Ac	9.19–45.00	1.41–178.33 *	9.78–285.00	118.90–255.00	3.50–44.80	21.67–54.80
mean ± SD (µg/100 g)		**28.03 ± 15.03 ^a^**	**112.83 ± 65.98 ^a^**	**181.6 ± 72.34 ^b^**	**174.45 ± 56.83 ^b^**	**25.41 ± 11.30 ^a^**	**36.64 ± 13.06 ^a^**
CV (%)		53.61	58.48	39.84	32.58	44.48	35.65
Min–max (µg/100 g)	Pha	11.10–19.80	1.41–318.33	23.83–170.00	7.70–295.00	13.33–68.30	18.33–94.70
mean ± SD (µg/100 g)		**11.64 ± 4.47 ^a^**	**193.19 ± 81.32 ^ab^**	**87.99 ± 53.31 ^a^**	**178.8 ± 74.75 ^b^**	**38.3 ± 21.37 ^a^**	**54.88 ± 34.66 ^a^**
CV (%)		38.39	42.09	60.58	41.81	55.80	63.17
Min–max (µg/100 g)	Bw	9.28–58.33	1.41–158.00	7.78–153.33	5.48–79.50	15.00–123.33	19.80–70.00
mean ± SD (µg/100 g)		**31.06 ± 18.11 ^a^**	**103.11 ± 62.94 ^a^**	**78.94 ± 47.02 ^a^**	**48.94 ± 29.88 ^a^**	**65.7 ± 29.23 ^a^**	**35.85 ± 19.01 ^a^**
CV (%)		58.29	61.04	59.56	61.06	44.50	53.03
Min–max (µg/100 g)	Li	10.41–36.90	1.41–281.67	90.1–240.00	9.20–271.67	100.00–554.00	18.85–2960
mean ± SD (µg/100 g)		**23.05 ± 13.85 ^a^**	**188.32 ± 101.04 ^ab^**	**139.46 ± 61.49 ^ab^**	**125.59 ± 72.38 ^ab^**	**280.15 ± 120.74 ^b^**	**1354.11 ± 883.19 ^c^**
CV (%)		60.11	53.65	44.09	57.64	43.10	65.22
Min–max (µg/100 g)	Ra	<LOD	1.41–156.67	93.5–275.00	9.78–350.00	16.67–44.70	19.67–80.00
mean ± SD (µg/100 g)			**75.94 ± 45.45 ^a^**	**125.92 ± 50.39 ^ab^**	**168.94 ± 98.49 ^b^**	**25.67 ± 8.07 ^a^**	**39.4 ± 25.85 ^a^**
CV (%)			59.86	40.01	58.30	31.43	65.62
Min–max (µg/100 g)	Gd	9.32–102.2	1.41–295.00	113.3–345	1.20–295.00 *	28.09–350.18	19.80–148.33
mean ± SD (µg/100 g)		**61.59 ± 36.22 ^b^**	**176.92 ± 114.54 ^ab^**	**203.26 ± 94.82 ^b^**	**158.53 ± 103.27 ^b^**	**196.31 ± 110.29 ^b^**	**84.38 ± 38.81 ^a^**
CV (%)		58.81	64.74	46.65	65.14	56.18	45.99
Min–max [µg/100 g]	He	8.91–68.33	1.41–606.67	125.00–185.00	150.00–328.33	28.51–231.67	553.33–986.67
mean ± SD (µg/100 g)		**46.43 ± 16.13 ^ab^**	**382.75 ± 178.9 ^b^**	**143.06 ± 18.97 ^ab^**	**231.04 ± 64.53 ^c^**	**141.33 ± 53.49 ^b^**	**669.86 ± 140.43 ^b^**
CV (%)		34.74	46.74	13.26	27.93	37.85	20.96
Min–max (µg/100 g)	Hd	8.19–720.00	1.41–420.00	8.28–275.00	7.78–410.00	28.21–256.67	19.50–136.67
mean ± SD (µg/100 g)		**394.55 ± 243.37 ^c^**	**199.69 ± 123.96 ^ab^**	**143.5 ± 86.66 ^ab^**	**221.29 ± 122.64 ^bc^**	**120.79 ± 74.09 ^b^**	**53.56 ± 34.74 ^a^**
CV (%)		61.68	62.08	60.39	55.42	61.34	64.85
H		29.366	28.445	23.412	26.564	55.846	39.034
p		<0.001	<0.001	0.001	<0.001	<0.001	<0.001

Ac—acacia honey; Pha—phacelia honey; Bw—buckwheat honey; Li—linden honey; Ra—rapeseed honey; Gd—goldenrod honey; He—heather honey; Hd—honeydew honey. Values marked with different letters are significantly different according to Kruskal–Wallis test and should be interpreted along the columns. LOD—limit of detection (µg/100 g): salicylic acid, 1.30; *p-*coumaric acid, 2.00; caffeic acid, 1.90; *trans-*ferulic acid, 1.70; vanillic acid, 2.00; vanillin, 1.30. * If the calculated content of the determined compound was lower than the limit of detection (LOD), a value of LOD/2^0.5^ was used.

**Table 3:** Content of flavonoids in Polish varietal honeys.

	Honey Variety	Pinocembrin	Hesperidin	Rutin	Quercetin	Hesperetin	Luteolin	Kaempferol	Isorhamnetin	Chrysin	Acacetin
Min–max (µg/100 g)	Ac	5.78–133.33	<LOD	33.33–135	<LOD	<LOD	<LOD	<LOD	21.21–150.00	5.05–91.67	7.78–150.00
**mean ± SD (µg/100 g)**		**85.49 ± 49.36 ^a^**		**76.88 ± 45.26 ^a^**					**105.09 ± 49.21 ^b^**	**60.83 ± 27.18 ^a^**	**95.28 ± 52.43 ^b^**
CV (%)		57.74		58.87					46.82	44.69	55.03
Min–max (µg/100 g)	Pha	7.70–286.67	43.04–326.67	46.67–568.33	<LOD	<LOD	<LOD	10.01–60.00	21.98–95.00	5.91–99.60	7.08–138.33
**mean ± SD (µg/100 g)**		**164.44 ± 84.69 ^a^**	**160.47 ± 121.79 ^b^**	**207.95 ± 136.1 ^b^**				**28.61 ± 17.73 ^a^**	**49.41 ± 27.47 ^ab^**	**63.07 ± 36.19 ^a^**	**74.92 ± 55.29 ^b^**
CV (%)		51.50	75.90	65.45				61.96	55.60	57.37	73.80
Min–max (µg/100 g)	Bw	7.65–158.33	41.84–260.00	46.07–148.33	4.95–50.10	15.00–40.00	<LOD	<LOD	<LOD	<LOD	<LOD
**mean ± SD (µg/100 g)**		**74.45 ± 50.58 ^a^**	**90.54 ± 56.06 ^ab^**	**79.06 ± 39.25 ^a^**	**19.9 ± 15.18 ^a^**	**19.83 ± 6.48 ^a^**					
CV (%)		67.93	61.92	49.65	76.31	32.67					
Min–max (µg/100 g)	Li	9.48–185.00	41.80–110.70	44.61–220.00	4.90–481.67	18.38–46.67	<LOD	10.61–53.33	20.21–56.50	4.95–88.33	7.48–25.00
**mean ± SD (µg/100 g)**		**79.11 ± 56.16 ^a^**	**60.16 ± 26.73 ^a^**	**117.33 ± 65.52 ^a^**	**223.38 ± 186.24 ^b^**	**26.03 ± 10.42 ^a^**		**15.52 ± 12.75 ^a^**	**31.80 ± 16.17 ^a^**	**49.98 ± 30.5 ^a^**	**13.83 ± 7.5 ^a^**
CV (%)		70.99	44.43	55.84	83.37	40.02		82.15	50.85	61.02	54.25
Min–max (µg/100 g)	Ra	7.74–225.00	42.81–1185.20	46.60–210.00	<LOD	<LOD	<LOD	9.65–53.33	21.64–76.67	1.56–130.00 *	6.98–106.67
**mean ± SD (µg/100 g)**		**94.72 ± 59.33 ^a^**	**560.33 ± 424.81 ^c^**	**139.33 ± 63.58 ^ab^**				**31.85 ± 16.38 ^a^**	**54.03 ± 21.98 ^ab^**	**59.49 ± 42.02 ^a^**	**83.22 ± 37.82 ^b^**
CV (%)		62.64	75.81	45.63				51.43	40.67	70.63	45.44
Min–max (µg/100 g)	Gd	7.08–130.00	40.80–81.70	45.72–105.00	<LOD	<LOD	<LOD	10.61–50.00	<LOD	4.85–55.00	<LOD
**mean ± SD (µg/100 g)**		**83.03 ± 49.85 ^a^**	**54.1 ± 13.84 ^a^**	**65.31 ± 22.64 ^a^**				**20.43 ± 15.77 ^a^**		**30.93 ± 22.73 ^a^**	
CV (%)		60.04	25.59	34.67				77.16		73.50	
Min–max (µg/100 g)	He	7.38–136.67	41.80–303.33	<LOD	<LOD	16.67–41.11	<LOD	<LOD	<LOD	5.11–60.00	7.71–31.00
**mean ± SD (µg/100 g)**		**81.81 ± 37.98 ^a^**	**174.51 ± 74.83 ^b^**			**23.29 ± 7.95 ^a^**				**40.10 ± 16.00 ^a^**	**18.51 ± 10.82 ^a^**
CV (%)		46.43	42.88			34.14				39.91	58.42
Min–max (µg/100 g)	Hd	7.78–250.00	43.64–351.67	38.33–170.00	4.65–52.60	18.30–75.00	8.49–80	<LOD	21.20–321.67	4.56–120.00	<LOD
**mean ± SD (µg/100 g)**		**86.15 ± 68.67 ^a^**	**126.5 ± 109.36 ^ab^**	**84.41 ± 48.84 ^a^**	**23.1 ± 19.91 ^a^**	**33.2 ± 23.37 ^a^**	**26.23 ± 21.82**		**128.02 ± 109.05 ^b^**	**40.01 ± 27.36 ^a^**	
CV (%)		79.71	86.45	57.85	86.22	70.39	83.19		85.18	68.37	
H		10.346	19.105	18.369	6.382	4.723	-	6.178	19.352	10.268	13.029
P		0.170	0.004	0.005	0.041	0.193	-	0.103	<0.001	0.114	0.011

Ac—acacia honey; Pha—phacelia honey; Bw—buckwheat honey; Li—linden honey; Ra—rapeseed honey; Gd—goldenrod honey; He—heather honey; Hd—honeydew honey. Values marked with different letters are significantly different according to Kruskal–Wallis test and should be interpreted along the columns. LOD—limit of detection (µg/100 g): pinocembrin, 1.30; hesperidin, 1.50; rutin, 1.20; quercetin, 2.00; hesperetin, 1.40; luteolin, 2.00; kaempferol, 1.40; isorhamnetin, 1.90; chrysin, 2.20; acacetin, 2.30. * If the calculated content of the determined compound was lower than the limit of detection (LOD), a value of LOD/2^0.5^ was used for calculations.

**Table 4:** Total phenolic content and antioxidant activity of the Polish varietal honeys.

Honey Variety	Total Phenolic Content (mg GAE/100 g)	Antioxidant Activity Toward the DPPH Radicals (%)
	Mean ± SD (min–max)	Mean ± SD (min–max)
Acacia (n = 8)	17.7 ± 2.8 ^a^ (11.6–20.9)	18.1 ± 1.8 ^a^ (16.1–21.6)
Phacelia (n = 13)	17.2 ± 2.3 ^a^ (14.3–21.2)	18.4 ± 1.5 ^a^ (16.1–20.8)
Buckwheat (n = 11)	123.5 ± 25.5 ^b^ (74.3–178.1)	91.7 ± 3.6 ^c^ (82.7–94.7)
Linden (n = 10)	33.3 ± 6.7 ^a^ (20.5–44.7)	35.7 ± 4.5 ^ab^ (28.0–42.5)
Rapeseed (n = 10)	26.7 ± 3.8 ^a^ (22.0–35.0)	31.7 ± 3.2 ^ab^ (26.3–37.9)
Goldenrod (n = 7)	31.0 ± 3.5 ^a^ (25.1–37.6)	25.1 ± 8.4 ^ab^ (13.3–38.4)
Heather (n = 8)	84.7 ± 9.5 ^b^ (69.6–96.7)	49.6 ± 10.8 ^b^ (38.3–70.1)
Honeydew (n = 17)	71.0 ± 16.7 ^b^ (44.0–101.3)	89.4 ± 6.6 ^c^ (74.8–98.1)

Values marked with different letters are significantly different according to Kruskal–Wallis test.

**Table 5:** Correlation coefficients between antioxidant activity and phenolic compounds, total phenolic content, pollen content, and electrical conductivity.

Variable	Correlation Coefficient with DPPH Radical Scavenging Activity	*p*-Value
**Salicylic acid**	**0.274**	0.012
*p-*coumaric acid	0.076	0.495
Caffeic acid	−0.101	0.361
*trans-*ferulic acid	0.048	0.666
**Vanillic acid**	**0.259**	0.018
Vanillin	0.015	0.890
**Pinocembrin**	**−0.261**	0.017
Hesperidin	0.080	0.471
**Rutin**	**−0.288**	0.008
**Quercetin**	**0.229**	0.036
Hesperetin	0.056	0.616
**Luteolin**	**0.281**	0.010
**Kaempferol**	**−0.269**	0.013
**Isorhamnetin**	**−0.285**	0.009
**Chrysin**	**−0.460**	<0.001
**Acacetin**	**−0.495**	<0.001
**TPC**	**0.894**	<0.001
Specific pollen content (nectar honeys)	0.120	0.333
**Electrical conductivity** **(honeydew honeys)**	**0.698**	0.002

Values in bold are significant at α = 0.05; TPC—total phenolic content; DPPH—antioxidant activity against DPPH radical.

## Data Availability

The original contributions presented in this study are included in the article and Supplementary Materials. Further inquiries can be directed toward the corresponding author.

## Supplementary Materials

The following supporting information can be downloaded at https://www.mdpi.com/article/10.3390/molecules30020360/s1, Figure S1: HPLC-DAD chromatogram of standards. The peaks correspond to the following: (1) vanillic acid; (2) caffeic acid; (3) vanillin; (4) *p-*coumaric acid; (5) *trans-*ferulic acid; (6) salicylic acid; (7) hesperidin; (8) rutin; (9) quercetin; (10) hesperetin; (11) luteolin; (12) kaempferol; (13) isorhamnetin; (14) pinocembrin; (15) chrysin; and (16) acacetin. Figure S2: Chromatogram of a rapeseed honey sample. Table S1: Eigenvalues and the proportion of variation (%) explained by the principal components (PCA) performed for all honey samples. Table S2: Correlations between the principal components and the original variables (PCA) performed for all honey samples. Table S3: Eigenvalues and the proportion of variation (%) explained by the principal components (PCA) performed only for nectar honey samples; Table S4: Correlations between the principal components and the original variables (PCA) performed only for nectar honey samples.
