# Supplementary material for: How Phenolic Compounds Profile and Antioxidant Activity Depend on Botanical Origin of Honey—A Case of Polish Varietal Honeys
Source: Molecules. 2025 Jan 17;30(2):360. doi: 10.3390/molecules30020360 (PMC11767671; doi:10.3390/molecules30020360)
Supplement: Supplementary file 1 [file molecules-30-00360-s001.zip › molecules-3401465-supplementary.pdf]

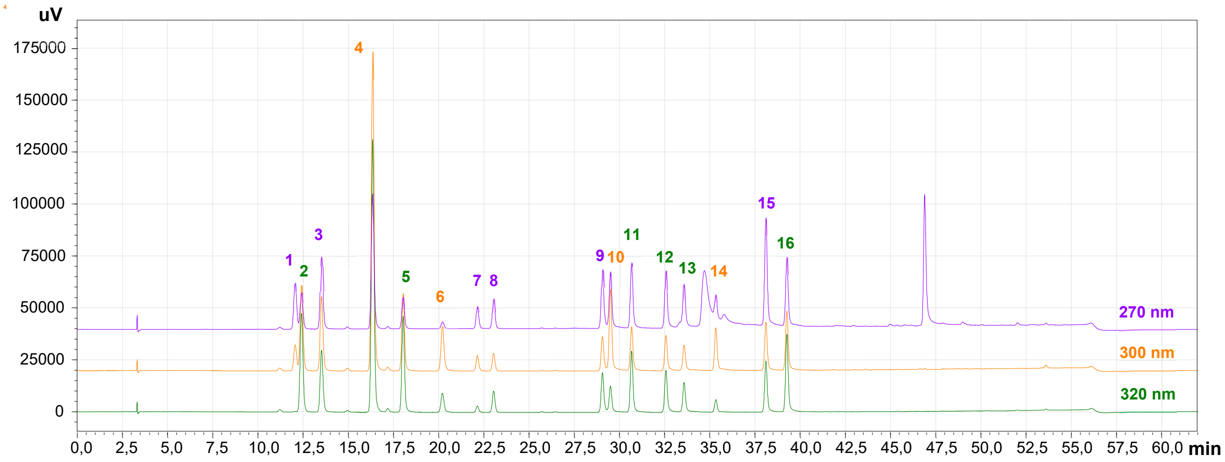

Figure S1. HPLC-DAD chromatogram of standards. The peaks correspond to the following: (1) vanillic acid; (2) caffeic acid; (3) vanillin; (4) p-coumaric acid; (5) trans-ferulic acid; (6) salicylic acid; (7) hespirdin; (8) rutin; (9) quercetin; (10) hesperetin; (11) luteolin; (12) kaempferol; (13) isorhamnetin; (14) pinocembrin; (15) chrysin; (16) acacetin.

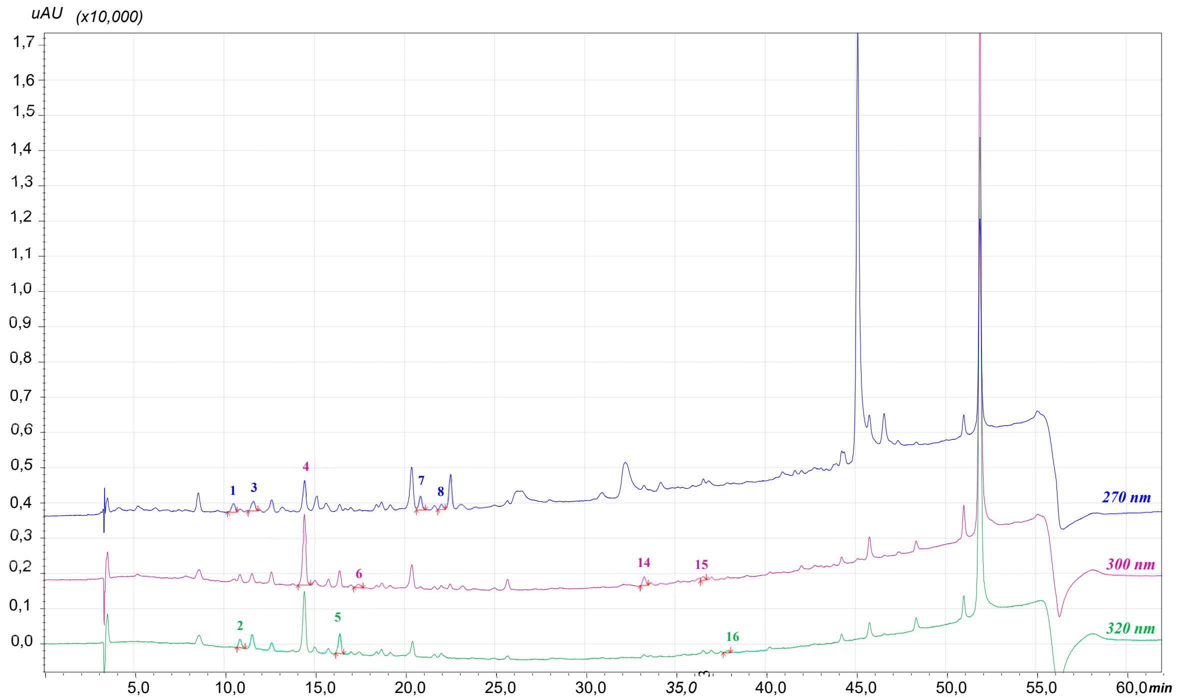

Figure S2. Chromatogram of a rapeseed honey sample

Table S1. Eigenvalues and the proportion of variation (%) explained by the principal components – PCA performed for all honey samples

Component	Eigenvalue	Proportion [%}	Cumulative [%]
1	3.32	18.46	18.46
2	2.78	15.45	33.91
3	2.05	11.41	45.32
4	1.60	8.89	54.20
5	1.35	7.47	61.67
6	1.26	7.01	68.69
7	1.05	5.82	74.51
8	0.90	4.98	79.48
9	0.83	4.59	84.08
10	0.67	3.71	87.78
11	0.52	2.86	90.65
12	0.39	2.16	92.80
13	0.32	1.80	94.60
14	0.30	1.68	96.28
15	0.27	1.49	97.77
16	0.24	1.31	99.08
17	0.10	0.55	99.62
18	0.07	0.38	100.00

Table S2. Correlations between the principal components and the original variables- PCA performed for all honey samples

Variable	Principal component						
	1	2	3	4	5	6	7
Vanillic acid	-0.21	-0.41	0.23	-0.49	-0.05	-0.05	0.06
Caffeic acid	0.07	-0.42	-0.36	-0.31	-0.57	-0.33	-0.16
Vanillin	-0.16	-0.07	0.88	-0.02	0.07	-0.34	0.04
P-coumaric acid	-0.19	-0.58	0.04	-0.36	0.32	-0.25	0.24
Trans-ferulic acid	-0.05	-0.70	-0.14	-0.22	0.24	-0.01	0.21
Salicylic acid	-0.48	-0.40	-0.35	0.46	0.05	-0.17	0.01
Hesperidin	0.48	0.19	-0.19	0.16	-0.10	-0.25	0.66
Rutin	0.44	-0.29	0.06	0.04	0.21	0.19	0.14
Quercetin	-0.26	-0.09	0.73	0.37	-0.06	-0.33	0.05

Hesperitin	-0.07	-0.42	0.03	0.04	-0.81	0.07	0.12
Luteolin	-0.41	-0.02	0.01	0.64	-0.14	0.00	0.05
Kaempferol	0.59	0.24	-0.11	0.07	-0.03	-0.31	0.42
Isorhamnetin	-0.02	-0.29	-0.40	0.21	0.25	-0.63	-0.35
Pinocembrin	0.24	-0.71	-0.08	0.28	0.19	0.34	0.06
Chrysin	0.33	-0.60	0.12	0.37	-0.04	0.24	-0.02
Acacetin	0.74	0.18	-0.17	0.09	0.08	-0.24	-0.23
TPC	-0.75	0.26	-0.25	-0.13	0.08	0.06	0.19
DPPH	-0.83	0.17	-0.34	0.10	0.05	-0.02	0.23

Table S3. Eigenvalues and the proportion of variation (%) explained by the principal components – PCA performed only for nectar honey samples

**Component**	**Eigenvalue**	**Proportion [%}**	**Cumulative [%]**
1	3.57	18.77	18.77
2	3.49	18.38	37.16
3	2.07	10.91	48.06
4	1.60	8.44	56.50
5	1.31	6.91	63.41
6	1.24	6.52	69.93
7	0.93	4.87	74.80
8	0.89	4.71	79.51
9	0.81	4.28	83.79
10	0.61	3.21	87.01
11	0.56	2.94	89.95
12	0.47	2.50	92.45
13	0.35	1.86	94.32
14	0.31	1.63	95.94
15	0.24	1.26	97.20
16	0.22	1.17	98.37
17	0.19	0.98	99.35
18	0.08	0.43	99.78
19	0.04	0.22	100.00

Table S4. Correlations between the principal components and the original variables- PCA performed only for nectar honey samples

Variable	Principal component					
	1	2	3	4	5	6
Vanillic acid	-0.15	0.49	-0.10	0.31	-0.47	-0.35
Caffeic acid	0.29	0.25	-0.41	0.51	0.31	-0.37
Vanillin	-0.35	0.35	0.73	0.17	-0.14	-0.19
P-coumaric acid	0.00	0.56	-0.07	0.01	-0.47	-0.23
Trans-ferulic acid	0.30	0.57	-0.19	-0.13	-0.35	-0.08
Salicylic acid	0.15	0.77	-0.09	-0.23	0.19	0.08
Hesperidin	0.42	-0.40	0.11	-0.35	0.08	-0.52
Rutin	0.45	0.11	0.06	-0.21	-0.25	0.03
Quercetin	-0.32	0.33	0.74	-0.03	0.25	-0.10
Hesperitin	0.13	0.50	-0.21	0.22	0.59	-0.34
Luteolin	-0.15	0.13	0.60	-0.10	0.14	-0.02
Kaempferol	0.43	-0.42	0.13	-0.12	0.01	-0.46
Isorhamnetin	0.54	-0.30	0.09	0.49	-0.06	0.23
Pinocembrin	0.51	0.57	-0.15	-0.39	0.03	0.23
Chrysin	0.50	0.54	0.10	-0.25	0.28	0.23
Acacetin	0.65	-0.48	0.12	0.21	-0.02	0.12
TPC	-0.77	-0.12	-0.39	-0.22	0.08	-0.04
DPPH	-0.76	-0.18	-0.33	-0.26	0.08	-0.06
Specific pollen content	0.33	-0.36	0.02	-0.55	-0.05	-0.29
